# The implications of Methylphenidate use by healthy medical students and doctors in South Africa

**DOI:** 10.1186/1472-6939-15-20

**Published:** 2014-03-04

**Authors:** Chad Beyer, Ciara Staunton, Keymanthri Moodley

**Affiliations:** 1 Centre for Medical Ethics and Law, Department of Medicine, Faculty of Medicine and Health Sciences, Stellenbosch University, Tygerberg Campus, Cape Town, South Africa

**Keywords:** Methylphenidate, Ritalin, Healthy, Medical students, Doctors, Utility, Cognitive, Enhancement

## Abstract

**Background:**

The use of medical stimulants to sustain attention, augment memory and enhance intellectual capacity is increasing in society. The use of Methylphenidate for cognitive enhancement is a subject that has received much attention in the literature and academic circles in recent times globally. Medical doctors and medical students appear to be equally involved in the off-label use of Methylphenidate. This presents a potential harm to society and the individual as the long-term side effect profile of this medication is unknown.

**Discussion:**

The implication of the use of Methylphenidate by medical students and doctors has not been fully explored. This article considers the impact of this use on the traditional role of medicine, society, the patient and suggests a way forward. We discuss the salient philosophy surrounding the use of cognitive enhancement. We query whether there are cognitive benefits to the use of Methylphenidate in healthy students and doctors and whether these benefits would outweigh the risks in taking the medication. Could these benefits lead to tangible outcomes for society and could the off label-use of Methylphenidate potentially undermine the medical profession and the treatment of patients? If cognitive benefits are proven then doctors may be coerced explicitly or implicitly to use the drug which may undermine their autonomy. The increased appeal of cognitive enhancement challenges the traditional role of medicine in society, and calls into question the role of a virtuous life as a contributing factor for achievement. In countries with vast economic disparity such as South Africa an enhancement of personal utility that can be bought may lead to greater inequities.

**Summary:**

Under the status quo the distribution of methylphenidate is unjust. Regulatory governmental policy must seek to remedy this while minimising the potential for competitive advantage for the enhanced. Public debate on the use of cognitive enhancement is long overdue and must be stimulated. The use of Methylphenidate for cognitive enhancement is philosophically defendable if long-term research can prove that the risks are negligible and the outcomes tangible.

## Background

Methylphenidate (Ritalin, Concerta) has been used since 1960 for treating children and adults suffering from attention deficit hyperactivity disorder (ADHD). It acts by delaying the reuptake of the neurotransmitters nor-adrenaline and dopamine, which prolongs their biochemical effects in the central nervous system. This has been shown to result in an increase in attention and a decrease in restlessness in children and adults who have been diagnosed with ADHD [1]. It has also been proven to significantly improve the ability to stay awake in patients suffering from sleeping disorders such as narcolepsy [2]. Perhaps desiring similar effects, the prevalence of Methylphenidate usage for non-medical cognitive enhancement purposes by students and academics alike is increasing on university campuses worldwide [3,4]. Medical campuses appear to be no different. After graduating high school in South Africa prospective medical students enter a 6 year degree of MB, ChB (Bachelors in Medicine, Bachelors in Surgery) followed by two years of internship and one year of community service before being registered with the Health Professions Council of South Africa as medical practitioners. The two years of internship and one year of community service are often done in rural settings, where the patient to doctor ratio is high. Arguably with the duration and intensity of the coursework and the long hours expected of medical students and interns within South Africa, the levels of illicit use may even be higher [5]. While the benefits of Methylphenidate in patients suffering from attention deficit disorders have been tested and proven, the benefits for a healthy person cannot be assured. There are a number of concerning studies that have emerged recently which illuminate the needs for long-term prospective studies on the use of methylphenidate amongst the healthy, as well as public discourse on cognitive enhancers in general and methylphenidate in particular. These studies suggest that the use of methylphenidate by the healthy may result in a cognitive trade off of enhancement of executive function in novel tasks with impairment of previously established performance. Additional studies have supported this idea, showing that although healthy users of methylphenidate may perceive an increase in current aptitude, they may actually be losing their general aptitude. Furthermore, in sleep-deprived individuals treated with Methylphenidate, results have shown that people overestimated their own performance, and experienced a subjective sensation of stimulation with only a mediocre improvement in attention. The perception of methylphenidate as an effective cognitive enhancer may be more related to the subjective effects experienced by the user than its realistic implications. This subjectivity contributes to the common human tendency of overestimating new technologies in the discussion of their benefits and risks [6]. As medical practitioners, it is of utmost importance to use evidence -based medicine to inform best clinical practice, hence it would be incorrect to extrapolate the effects of Methylphenidate for the healthy from the evidence that stands for those with medical indications [7]. The evidence of the effect of Methylphenidate on healthy people is lacking, not just quantitatively, but in reliability. The latter is mostly due to the relative lack of comparative baseline variables being tested, such as not attempting to, or not being able to quantify the baselines for intelligence, wakefulness or cognitive ability in the subjects tested. Furthermore, of the available studies, the majority are single dose studies, although it has been shown that Methylphenidate follows dose dependent nonlinear kinetics in narcoleptic and healthy patients [8]. This means that the effects of an increase in dose cannot be accurately predicted on the basis of a single dose study. As a large number of the studies of Methylphenidate’s efficacy and therapeutic safety profile have been conducted on children, the safety and efficacy of Methylphenidate may vary in unforeseen ways between adult medical students, practitioners and the children on whom these studies were conducted. It is therefore critically important to acquire further evidence regarding the quantified and dose dependent effects on differing groups of healthy patients before any decision can be made. It is time for ‘medicine, healthcare and society to prepare for broader and more prevalent non-medical uses of pharmaceuticals’ [3] and to debate all the implications thereof.

## Discussion

### Methylphenidate: efficacy and adverse effects

Systematic review has shown that the expected effects of methylphenidate exceed its actual effects, as demonstrated in single or double-blind randomised controlled trials [9]. A single dose of methylphenidate has been shown to benefit memory in healthy subjects, as well as increase subjective ratings for interest, excitement and motivation in a mathematical task. This effect was not found in passive tasks. Indeed there is evidence that usage of these substances by patients without an attention deficit disorder may result in a cognitive trade off [7] by increasing their short term memory at the expense of their long term memory. The perception of Methylphenidate as a major drug of enhancement may be more closely linked with the association of educational success in school children. The idea of this association leads to the suggestion of the potential for an enhanced level of cognitive functioning in the healthy. However, educational success is better understood as being attributed to multiple factors [10]. This presents a confounding factor in neuropsychological research, in that a person’s performance in research tests is influenced by more than one cognitive process, since these processes are not isolated, but rather work together and are interrelated [11]. This interwoven nature of cognition renders it difficult to assess specific avenues of cognitive enhancement by only considering the efficacy of a person performing novel cognitive tasks.

Unlike a drug intended to cure or remedy a condition; wherein the efficacy can be quantified on inhibition of an organism (in terms of antibiotics) or improvement to a normal level of functioning (in terms of antipsychotics), the testing of whether Methylphenidate adds to an already functional person’s utility may be difficult to quantify. How would a researcher be able to adequately assess the varying baselines of functionality and the relative subjectivity of human traits like intelligence, concentration and aptitude? Perhaps concentration could be shown to be qualitatively enhanced. However, at what level of qualitative analysis would we find sufficient evidence to deem the drug effective and more so than conventional means of concentration and wakefulness enhancement, such as a good night’s sleep or a strong cup of coffee? It is important to exclude confounders such as these in the studies conducted on Methylphenidate use in the healthy, for we cannot correlate Methylphenidate use and any cognitive outcome on the user without all of these co-founding variables being accounted for.

### The role of medicine in cognitive enhancement

The conventional role of medical intervention is that of curing illness and restoring the patient to a functional or ‘normal’ level. Further consideration would be necessary as to whether utilitarian ethics justifies or vilifies supporting research into medications intended to enhance the human condition, rather than medications which support this conventional role of medical intervention. Perhaps it could be argued that an enhancement of perceived aptitude would aid in mental wellbeing, but medical practitioners need to be wary of walking the tenuous line between treating real illness and over treating perceived inadequacy. The usage or advocation for usage of cognitive enhancement medication may necessitate a re-evaluation of the role of medicine in society. However, it can be argued that the concept of Methylphenidate as an enhancer does not represent a challenge to the historical view of the role of medicine. The use of, for example, laser surgery to correct visual abnormalities often returns the person to a level of vision that is greater than their baseline and enhancement is used and advocated for on a widespread basis when we take vaccines to prepare our immune response against certain infectious diseases [12]. Therefore the correlation between illness and intervention may never have been as clear. Perhaps enhancement is more congruent than incompatible with the current role of medicine in society.

The widespread use of Methylphenidate for enhancement purposes requires a scrutiny of the cost-benefit analysis of medical intervention for the patient. Medical intervention is usually justified by the analysis of the utility lost under a medical condition justifying the possible adverse effects of that intervention. This is why a patient suffering from cancer may be justifiably treated with radiation therapy, although the adverse effects may be appreciable. However, a medical practitioner would find it difficult to justify such a radical intervention for a less fatal condition. This analysis is particularly relevant for Methylphenidate, as the identified side effects may not be acceptable or ethically justifiable for a patient who does not have a condition that justifies them. Some of the most frequently encountered side effects are a decreased appetite, dry mouth, sleeping problems, mild forms of depression and repetitive movements (tics) [13]. More dangerous side effects associated with Methylphenidate use involve an exacerbation of predisposed conditions such as anxiety disorders or of pre-existing conditions such as cardiac arrhythmias and glaucoma. These major side effects and contra-indications for Methylphenidate usage maybe ameliorated if a medical practitioner thoroughly investigates his/her patient and rules out dangerous physiological predispositions before prescribing the drug. Furthermore, continuous monitoring and the scheduling of Methylphenidate may assist in preventing and detecting cases of addiction, this would act to ameliorate the possibility that Methylphenidate even in the prescribed oral form, may have large abuse potential [14]. However, it is important to note that the use of Methylphenidate has been implicated in lowering, not elevating, the chance of future abuse of stimulants [15]. Very little is known about long term use in the healthy, and the widespread neurochemical systems implicated in the use of Methylphenidate suggest that in addition to cognition they may impact emotional and motivational functions [16]. A further risk is that serious side effects like cardiac arrhythmias have a higher likelihood amongst older people with incipient cardiovascular disease and that this group of people and older doctors are increasingly likely to use the drug to stave off the fraying of their cognitive ability [17].

Under the status quo, wherein people acquire Methylphenidate either from a colleague or another illicit source, these risks predispose them to unforeseen and potentially fatal side effects [1]. This presents a far greater risk to the public compared to scheduled, controlled, medically moderated distribution of the medication. It is true that Methylphenidate has been declared safe enough for widespread use in children and young people with ADHD for a long period of time. As ADHD is not life-threatening, an analogy may be drawn with the benefits of therapeutic intervention being similar to those of enhancement intervention. The same benefits would seemingly justify its use from a safety perspective as well, as the adults using Methylphenidate for enhancement purposes likely value these effects as much as those with ADHD. In addition to this, Methylphenidate has been proved to have a low enough risk profile to be ethical for use in research to test cognitive effects in healthy subjects, where the effect is clearly more elective than therapeutic [18]. The societal sense of unease with cognitive enhancement could be seen as analogous to the early stages in the development of cosmetic surgery. What was, for a time, seen as the patient irrationally accepting a large risk for what was perceived to be a small, unnecessary benefit, has grown to be a relatively accepted form of self-augmentation, which increases the general happiness of the patient, inherently a good thing. Furthermore the history of cosmetic surgery shows that many medical practitioners, given the right evidence and cultural frameworks, may become comfortable with non-therapeutic interventions [19]. Cognitive enhancement medication may follow a similar path [20].

### Philosophy of cognitive enhancement

The fields of philosophy and bioethics surrounding cognitive enhancement have provided many debates and differing stances [17,18,21]. The intention in this section is to summarise the salient points of cognitive enhancement in general, and then to analyse how these points tie in with the idea of medical students and doctors using Methylphenidate.

The arguments surrounding augmenting the human condition draw a strong analogy with the ethical debates surrounding gene doping in sport. This involves the use of biochemistry to alter the expression of certain genetic sequences in human genotypes leading to the expression of desirable, or inhibition of undesirable, cells or human genes to improve athletic performance. The primary argument against this is that it presents an actual or potential health risk to the athlete [22] which ties strongly with the medical principle of *primum non nocere* (first do no harm). The second argument against genetic doping is that it violates the spirit of sport in lieu of the principles of ethics, honesty and respect for rules governing the profession [22]. These values, although tenuous in sport, are certainly applicable in the medical profession.

There is, however, far less consensus on what constitutes cheating in a medical environment. If Methylphenidate usage amongst the healthy does provide practical benefits for the user and these benefits actively translate into tangible outcomes then it is easy to consider the example of a medical student who may achieve better marks, and potentially pursue a more competitive speciality by using Methylphenidate to gain a competitive advantage over their peers [23]. In this example there is a demonstrable harm upon the unaugmented students, as they have been placed at a comparative disadvantage in what could be argued to be an unfair manner.

The lack of consensus on what constitutes cheating becomes more prominently an issue for the sporting analogy when dealing with cognitive enhancement amongst medical doctors and researchers. In contrast to the above scenario wherein a medical student may take the drug explicitly for personal academic success, it may be defensible to have our top medical researchers on cognitive enhancement drugs [24]. This is because research can be viewed as a non-zero sum game, wherein the potential achievements of an individual or group, such as the discovery of a cure for a disease such as HIV would surely benefit humankind far more than the individual doctor or academic. Another example is if an augmented doctor is able to concentrate to a greater extent on their patient and be more conscientious, it would likely lead to a greater outcome for the patient. In these two scenarios the benefits to the immediate doctors or researchers, and perhaps their comparative advantage over their colleagues [25], is not the only, or the most relevant, factor. The primary aim of the medical field should aim to do what is best for the patient and with this analysis any competitive advantage over colleagues appears to be more of a secondary outcome.

There is a further consideration in this calculation, that in improving relative inequalities and enhancing personal competencies we may begin to undermine the ability of the population at large to identify with the human characteristics of these athletes and academics. This may lead to their achievements being considered products of science rather than of human endeavour. This would invalidate the correlation with hard work, character, effort and achievement [26] which is one of the reasons why, for many years, cognitive enhancement has been considered incompatible with virtue ethics.

The pursuit of the good life is considered to be the pursuit of a virtuous life. This has led to the criticism of virtue ethics as being too demanding and thus obtainable only by an elite group of people [27]. This criticism has received support by studies in the behavioural sciences, which suggest that humans are cognitive constrained. Examples of these constraints are the findings that suggest humans are particularly bad at deferring judgement and making accurate decisions based on information given [28]. This undermines the pursuit of a good life in virtue ethics as normative theories are largely based on intuitions.

Joshua Green published a study in 2001 analysing what parts of the brain are involved in specific ethical reasoning [29]. He found that “deontological moral judgements are primarily driven by automatic emotional responses”. Furthermore, the stability of our judgements has also been found to be prone to manipulation through a vast array of methods, from hypnosis to transcranial magnetic stimulation. These findings, in collaboration with those previously discussed may suggest that the failings of the majority in the pursuit of eudaimonia are due to biological factors more so than inadequate training or motivation [27]. This renders the majority of people unable to achieve a good life due to factors beyond their control. Perhaps more importantly these findings also suggest that the way a person deals with moral dilemmas may be biologically constrained, open to manipulation and unreliable [30].

Recent articles have suggested that the use of cognitive enhancement as a facilitator, in adjunction with moral and epistemic virtues, may make the pursuit of a virtuous life, with morally responsible judgements, more achievable [31]. It is especially important to incorporate these virtues, as without them there can be no suggestion that cognitive enhancement would translate to moral goodness. This analysis is particularly important in the context of this article, as doctors, perhaps more so than other people, are ultimately reliant on the pursuit of a virtuous life. If cognitive enhancement helps to facilitate this it would certainly be an attractive outcome.

If we could ascertain that the use of Methylphenidate creates more effective doctors, the benefits to society are relatively self-evident. Furthermore, if this increased utility correlated to an acceleration of medical breakthroughs, and a potential increase in collective happiness, then this would satisfy Jeremy Bentham’s consequentialist basis for his Utilitarian moral philosophy [32], which states that the moral outcome is the one which leads to the greatest good for the greatest number. Opponents of this utilitarian approach to cognitive enhancement often take a deontological defence arguing that the act of using a drug to enhance your personal cognition may be hedonistic and thus not justifiable. However, Kant also argued that the categorical imperative could be interpreted through reason alone to delineate what inviolable rights are [33]. If we accept that the inherently inviolable right in this debate is that of the right to enhance personal utility then attempts to achieve that enhancement could be justifiable, regardless of an increase or a decrease in personal utility or societal benefit or detriment as an outcome, so long as the usage is duty driven.

The latter part of this argument is an important one, and requires comprehensive review by governmental bodies to prevent exploitation, either by the person who argues personal utility for societal benefit, or the society that may implicitly coerce such a person into pursuing this potential collective benefit at expense of their own autonomy. Rawls’ ‘veil of ignorance’ expansion places upon us an imperative to equalise distribution of resources to lessen our own risk of being born into oppression [34]. Avoiding potential exploitation may seem to invalidate the usage of Methylphenidate under a scenario of unfair advantage; however Rawls argued that we may accept unequal distributions under one condition: the inequality must be to the benefit of those who are the worst off [34]. Inequality in potential achievement for the sake of innovation and societal advancement may pass this analysis and satisfy the Rawlsian difference principle.

The salient point is that under the status quo the distribution of Methylphenidate for cognitive enhancement is, by the principle of equal liberty, unjust. Access to, and usage of, this drug amongst the healthy is restricted to a privileged class of people who are able to acquire it through their own personal, financial or social resources. This unregulated distribution further entrenches social inequality within a society, by restricting access for the less privileged and with no solid governmental policy moderating the access for the privileged. The following section will further advance the case for, and discuss the steps to implement, such a policy, while considering the implications thereof.

### The implications of, and policy for, allowing for moderated cognitive enhancement

If we extend the harms and risks inherent in the currently unregulated system of cognitive enhancement medications to a system when cognitive enhancement is endorsed, but remains unregulated [35], then there are various role-players we need to exam. Firstly, the individuals within society may be explicitly or implicitly coerced into using these medications due to utility calculations of corporate entities [36]. For example, a transport company, which is able to coerce their drivers to take medications to enhance wakefulness and concentration, would be in a position to transport goods further, with fewer stops, and thus be in a position to make a larger profit, than their competitors. This would give them a competitive advantage in their industry and their competitors would either have to adopt the same policy or face the prospect of being driven out of business. It may be an idealistic view that the medical community would be exempt from being drawn into methods of productivity enhancement.

This argument could be made with private medical facilities, having fewer doctors working longer hours during a call rotation would provide a profit motive for coercion by the administrators of these facilities [37]. Also, in many parts of the world the incentive [38] or requirement for doctors to work overtime in the medical sector is immense [39]. Coffee and energy drinks may make way for more effective methods of concentration and wakefulness enhancement. The principles of personal autonomy tend to support the idea that societal interference in these scenarios needs to be adequately justified lest it be deemed paternalistic, arbitrary and not legitimate. However, in an absolutely unregulated environment, as described above, the potential for coercion may violate the autonomy and rights to equality of the average, as choosing to not, or not being in a position to, enhance may put you at a competitive disadvantage [40]. This is because if we are to accept an inherent advantage provided by access to and usage of these drugs we may logically extend this advantage to an increase in employability for the augmented over the drug free.

In this way, if cognitive enhancement becomes only a pursuit of private persons and businesses, the public sector and the worse off may become less competitive. This is an especially relevant danger in South Africa where inequality in access to pharmaceuticals is drawn along an economic, and historically, a racial divide. This may lead to perpetuation of the economic divide, making the poor and previously disadvantaged even less able to gain employment and develop skills [41].

This calls for government to adopt an active policy to offset these potential harms, and allow equitable distribution of these resources in a society, or minimise the comparative advantage they provide. The former would require the development of generics and government subsidisation, increasing the availability of these drugs for the historically less privileged, an approach that would require state funding. Justice in equal distribution of resources in a medical setting would suggest that only after all legitimate medical concerns are adequately addressed within a population may state financing of enhancement medication be permissible. This approach, therefore, seems economically unfeasible.

Fortunately, when it comes to democratic societies, the state does not have to decide to either subsidise the development of, or illegalise a substance. They may act to curtail the use of these potentially hazardous drugs, whilst maintaining the autonomy of the individual, and promoting equitable distribution of resources. This is premise for the policy we will advocate for. This policy would include disincentivising people from using these drugs by educating the population on their realistic effects and relatively unknown side effect profile; while discouraging their usage by taxation included in the price of sale, as currently legal risky behaviour [35], like tobacco, is currently taxed.

The funds gained by the personal taxation of Methylphenidate could be allocated to the medical care of the most disadvantaged in society as well as the development of affordable generics when the long-term effects of these medications are known. An additional corporate tax on businesses whose employees use these medications would serve two purposes. Firstly it would increase the cost and therefore minimise the profit incentive of businesses coercing their employees into using these drugs, ameliorating this risk to personal autonomy. Secondly this new revenue stream for the state could be invested into the research on orphan disease, which are diseases that are so rare that there is no motive for private pharmaceutical companies to develop treatments for them. With this increased revenue the state could pursue this research [36].

If cognitive enhancement may be considered a new technology with potentially hazardous effects for those engaging in it, the state may require users to prove adequate knowledge of the enhancer by passing a test and acquiring a license, allowing for the capacity of informed consent. Furthermore, those that acquire this license could be compelled to acquire additional, appropriate medical insurance, to lessen the potential burden on the state. The people who are licensed for the use of these cognitive enhancers could be compelled to participate in a prospective cohort study on the long-term effects of these cognitive enhancers. Although the state may maintain their position on the potentially harmful and currently unknown effects of their long term use, citizens could maintain their autonomy and opt into the use of these enhancers, with all the above considerations [36], if they are able to make an informed decision on the potential risks involved. So the discouraged and disincentivised, but widely available status of cognitive enhancers such as Methylphenidate would: decrease the capacity for coercion, decrease the appeal of competitive advantage, provide financing for medical care of the most disadvantaged in society, and fund research into treating previously neglected conditions. This appears a better state of affairs than the status quo of the unregulated use of Methylphenidate.

We would further propose that medical students and doctors are a well-suited group to conscientiously opt into the use of cognitive enhancements for a multitude of reasons. These include the ideas that: they are more likely to understand the effects of these cognitive enhancers and less likely to be swayed by the exaggeration of their effects by pharmaceutical companies, they would be in a better position to understand the side effect profile of these enhancers, as well as the prospective long term effects, they would be more likely to provide accurate data during a long term prospective cohort study; they would be in a better position to access the additional medical insurance required in this policy; finally, once the research is conducted and results acquired the doctors involved in the study would be in a well informed position to advise public policy and debate on the profile of these cognitive enhancers.

There are further considerations specific to the individual role-players, including the patient, that still need to be accounted for. Allowing medical students and doctors free access to Methylphenidate may lead to some conducting the majority of their theoretical and clinical training under its influence. Which may distort their ability to accurately discern their level of competence without the drug, rendering them dependant on the drug to maintain confidence in providing adequate patient care [5,37]. This potential for abuse of Methylphenidate when used for cognitive enhancement may translate to a need to revise the scheduling of the drug [14]. Adjusting the dose of Methylphenidate for cognitive enhancement, marketing it as a different medication than when it is used for its traditional purpose and initially scheduling the medication for cognitive enhancement as a controlled substance, like morphine, would make moderation of distribution significantly easier. This would also greater enable the state regulating bodies of the medical profession, such as the Health Professions Council of South Africa, to accurately monitor the distribution of drugs explicitly for cognitive enhancement, ameliorating their specific risks for abuse, self-prescription and other areas of possible exploitation.

## Summary

The effects of Methylphenidate as a significant cognitive enhancer are more subjective than practical and the existing studies are insufficient to directly answer the long-term side effect profile of this medication amongst the healthy. Prospective cohort studies must be prioritised to make accurate decisions on Methylphenidate’s use for cognitive enhancement. Under the status quo the distribution of Methylphenidate is subject to personal, social and economic advantage. This unjust distribution has a large potential for increasing the amount of economic and social disadvantage within society. Regulatory governmental policy must seek to remedy this while minimising the potential for competitive advantage for the enhanced. This will be achieved by disincentivising citizens from their use; while discouraging their use by personal taxation, the requirement of the acquisition of a license; .additional medical insurance, the participation in a long-term cohort study on their use; and a further corporate tax to act against the potential for coercion A public debate on the use of cognitive enhancement is long overdue and must be stimulated by new analysis and research. The use of Methylphenidate for cognitive enhancement has been shown to be defensible from: a utilitarian perspective in terms of enhancing personal utility for the potential good of society; a deontological perspective through the principle of autonomy as an inviolable right; virtue ethics where the pursuit of a virtuous life is suggested to be more achievable with the addition of cognitive enhancement; a perspective of justice. If long-term research can prove that the risks are negligible and the outcomes tangible then we would defend the regulated use of Methylphenidate by the healthy and believe medical students and doctors represent a good group to initially implement this policy.

## Abbreviations

ADHD: Attention deficit hyperactivity disorder; HIV: Human immunodeficiency virus.

## Competing interests

The authors declare that they have no competing interests.

## Authors’ contributions

CB wrote the original essay, conducted the literature review, reworked the essay into article format. CS reviewed the first draft of the article, discussed restructuring of the article and contributed to subsequent drafts of the paper. KM conceived of the idea for the Ethics Essay Writing Competition, guided the rewriting into article format, provided relevant literature, reviewed and edited all drafts of the paper. All authors read and approved the final manuscript.

## Authors’ information

CB is a medical student with a keen interest in Bioethics and Psychiatry. He originally wrote this article as an entry for the 2012 Ethics Essay Writing Competition sponsored by the Medical Protection Society (MPS) and organised by the Centre for Medical Ethics and Law for medical students at Stellenbosch University. The topic provided was ‘Why do healthy medical students use Methylphenidate?’ His was the winning entry and he received R5000 prize money at the MPS Annual Ethics Conference ‘Ethics for All’ hosted at the Cape Town International Convention Centre (CTICC) in November 2012. CS [LL.B (Civil Law), LL.M (Public Law)] is a PhD candidate in the Regulation of Stem Cell Research in Ireland from the National University of Ireland, Galway. She has served as an intern at the World Health Organisation, Geneva, Switzerland; as an Intern at the Centre for Bioethics and Law at the University of Stellenbosch, South Africa; a Legal Researcher of the Law Reform Commission of Ireland, Dublin, Ireland; a Legal Researcher on the Rape Attrition Project at the National University of Ireland Galway, Ireland; she is a part-time lecturer at the National University of Ireland Galway; is the recipient of various grants including the Irish Research Council Postgraduate Scholarship; has published various articles within the Medico-legal field; written various reports; presented various conference papers; and serves on the Board of Directors of the Irish Stem Cell Foundation in the capacity of legal advisor to the foundation. KM [MB ChB, MFamMed, MPhil (Applied Ethics), FCFP (SA), DPhil] is the Associate Professor and Head of the Centre for Medical Ethics and Law (CMEL) at Stellenbosch University. She is recognised as a leading authority in the field of Bioethics and has served on South Africa’s National Health Research Ethics Council (NHREC) in Pretoria. She is also a member of the following: the HIV Preventive Research Data Safety Monitoring Board (DSMB) in the Division of AIDS, National Institutes of Health (NIH), Washington DC, USA; the Scientific Review Committee, European and Developing Countries Clinical Trial Partnership (EDCTP), The Hague, Netherlands; the Board of Directors, South African Medical Research Council (MRC), Cape Town; SAGE Working Group on Immunisation in Humanitarian Crises, World Health Organisation (WHO), Geneva, Switzerland.

## Pre-publication history

The pre-publication history for this paper can be accessed here:

http://www.biomedcentral.com/1472-6939/15/20/prepub
